# Kaposi's sarcoma of the hand mimicking squamous cell carcinoma in a woman with no evidence of HIV infection: a case report

**DOI:** 10.1186/1752-1947-2-213

**Published:** 2008-06-19

**Authors:** Christophoros Kosmidis, Christopher Efthimiadis, Georgios Anthimidis, Georgia karayannopoulou, Marios Grigoriou, Kalliopi Vassiliadou, Eleni Berovali, Panagiotis Fachantidis, Epaminondas Fahantidis

**Affiliations:** 1 Department of Surgery, Interbalkan European Medical Center, Thessaloniki, Greece

## Abstract

**Introduction:**

Kaposi's sarcoma is a vascular neoplasm mainly affecting the skin of the lower extremities. Although it is the most common neoplasm affecting patients with AIDS, sporadic cases in HIV-negative people have been reported. It is a lesion mainly affecting men and its clinical presentation presents a challenge, as it can resemble other benign or malignant skin lesions.

**Case presentation:**

We report a rare case of Kaposi's sarcoma presenting in a 68-year-old Mediterranean woman with no evidence of HIV infection. The patient had a 6-month history of a slowly progressing pigmented lesion on the dorsum of her left hand. The lesion clinically resembled a squamous cell carcinoma. The patient was treated with a wide excision of the lesion and primary reconstruction with a full thickness skin graft. Histopathological and immunohistochemical analysis of the excised lesion revealed the presence of Kaposi's sarcoma. Serologic investigation for HIV was negative but polymerase chain reaction for human herpes virus type 8 infection was positive. Thorough clinical and imaging investigation of the abdomen and chest were both negative for loci of disease.

**Conclusion:**

Kaposi's sarcoma, although rare in its sporadic form, should be considered in the differential diagnosis of indeterminate skin lesions, especially those affecting the extremities.

## Introduction

Kaposi's sarcoma (KS) is an angioproliferative skin lesion associated with a great number of epidemiologic and pathophysiologic factors. Due to this variability it is classified into four distinct clinico-epidemiological types: classic Mediterranean KS, African-endemic KS, immunosuppressive drug-related KS and epidemic AIDS-related KS. Despite being the most common neoplasm affecting patients with AIDS, its sporadic presentation is rare and can sometimes escape clinical suspicion.

In its classic-sporadic type, KS presents as a cutaneous lesion typically affecting the skin of the extremities. Epidemiologically it is most often observed in elderly patients but it is a rare occurrence in females. The incidence is higher in Southern and Eastern European countries and the condition is more commonly found in Jewish, Italian and Greek populations.

## Case presentation

**Figure 1:**
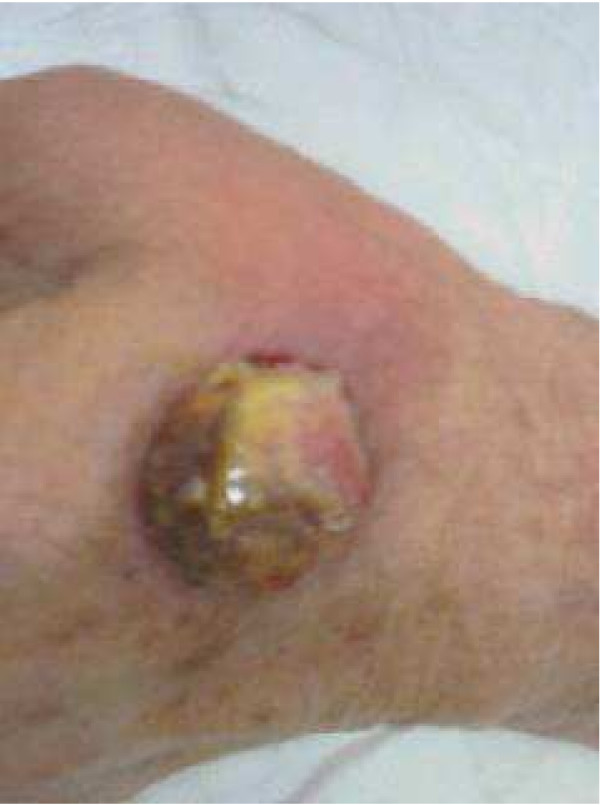
A primary Kaposi's sarcoma on a hand, which was first regarded as a squamous cell carcinoma.

**Figure 2:**
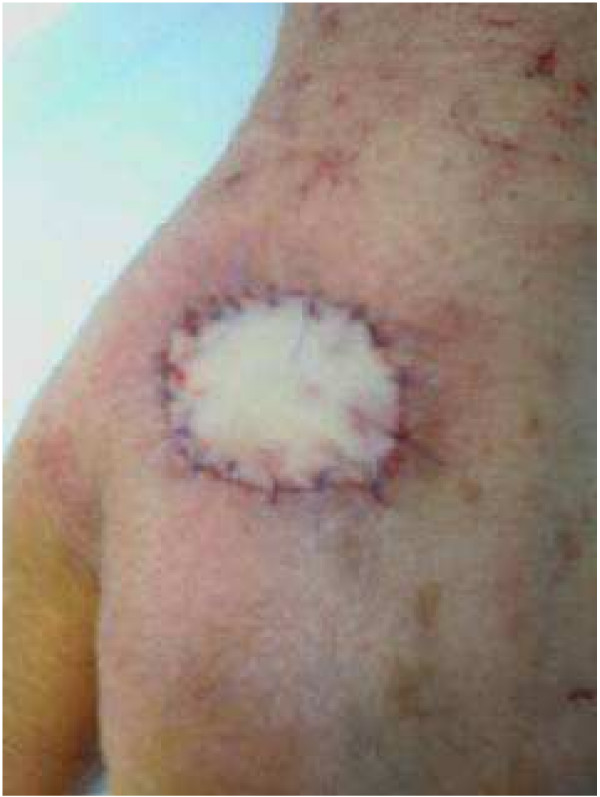
A wide and deep total excision of the lesion was performed and a full-thickness skin graft was used to cover the wound surface.

A 68-year-old woman presented with a 6-month history of a slowly evolving, asymptomatic, raised, slightly pigmented skin lesion, measuring 25–30 mm in diameter, involving the dorsum of her left hand (Figure 1). Cutaneous examination was otherwise normal. She was in otherwise good health, with no predisposing factors or conditions requiring medication. Clinically, the lesion resembled a squamous cell carcinoma. Due to the size and presentation of the lesion, a wide local excision of the skin and underlying subcutaneous tissues was performed. A full-thickness skin graft was used to reconstruct the excisional defect, providing an excellent aesthetic result (Figure 2). The donor site was the anterior surface of the left forearm, which was closed primarily.

**Figure 3:**
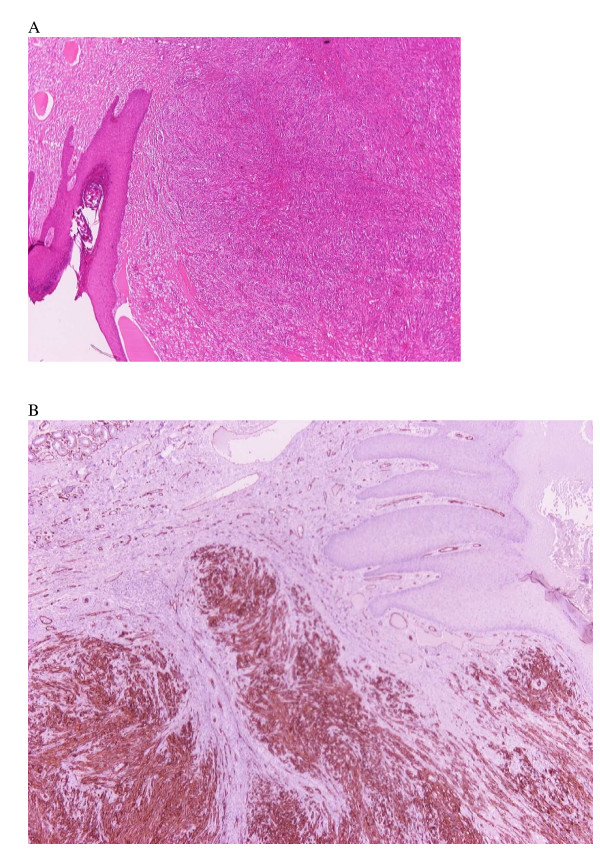
**A**) Kaposi's sarcoma consisting of angiomatoid vascular spaces and abundant spindle-shaped cells HE × 40. **B) **– Tumor cells are strongly positive for CD34 × 40.

The surgical specimen was submitted for pathological evaluation. Histological and immunohistological findings were consistent with a diagnosis of KS (Figure 3A and 3B). Serologic testing for HIV infection was negative, but the associated human herpes virus type 8 (HHV8) was detected by polymerase chain reaction (PCR) on the tissue samples.

Following the diagnosis the patient was subjected to a thorough diagnostic evaluation to determine the possible spread of the disease to other sites. Chest X-ray, abdominal ultrasonography, upper and lower gastrointestinal endoscopy, as well as thoracic and abdominal computed tomography, were all negative for the presence of disease.

Wide local excision with histologically negative margins is regarded as the accepted method of treating minimal cutaneous lesions of KS. Since no dissemination of the disease was demonstrated in the postoperative clinical and radiological evaluation, no further treatment modalities were considered necessary. During a 9-month follow-up, no local or distant recurrence was observed. Re-evaluation for HIV seroconversion was negative.

## Discussion

Since its first description, KS has remained a tumour of undetermined pathogenesis. There is still doubt regarding the nature of the proliferating cells and whether the lesions represent an exuberant hyperplasia or a neoplasm. KS lesions have a number of peculiar characteristics, including a lack of aneuploidy and a strong association with immunodeficiency. Emergence of KS in transplant recipients and immunosuppressed patients is remarkable because it may regress spontaneously if immunosuppression is reduced or discontinued. These characteristics suggest that it is not a malignant neoplasm but a benign, potentially controllable and reversible hyperplasia [1,2]. On the other hand, the lesion contains spindle cells that share features with endothelial cells and smooth muscle cells and are most likely primitive mesenchymal cells that can form vascular channels. These cell proliferations are monoclonal in origin, even in patients with multicentric lesions, indicating that KS is indeed a neoplasm [3].

The pathogenesis of KS is still under investigation. Current studies have focused on the search for a causative infectious agent mainly due to its correlation with immunocompromised patients. HHV8 infection is widespread in Mediterranean areas with a relatively high incidence of KS (Greece, some Eastern countries, Southern Italy, Sardinia, and Sicily). A large body of evidence has strongly linked all KS clinical variants with HHV8 infection [4].

HHV8 DNA is present in all forms of KS but not in other mesenchymal tumours or non-specific inflammatory lesions of the skin, suggesting a strong association of HHV8 and KS [5]. Moreover, non-lesional tissue samples from KS patients may harbour HHV8. HHV8 is identified in early KS lesions, supporting the role of HHV8 in the pathogenesis of this disease. Furthermore, it has been demonstrated that replicating HHV8 codes for proteins able to control cell growth and that these can lead to the development of KS. In particular, this virus can give rise to the generation of viral monocyte inflammatory protein, viral interleukin 8 receptor and viral interleukin 6, promoting angiogenesis and replication of the spindle cells typical of KS [6,7].

The presence of HHV8 infection is now considered essential for the development of KS. However, the incidence of HHV8 infection is far higher than the prevalence of KS, suggesting that viral infection per se is not adequate for the development of malignancy and that one or more cofactors are necessary. The exact interactions of HHV8 infection with other factors known to be involved in the pathogenesis of KS, including anti-apoptosis genes and cytokines, which may be mediated by virally encoded genes as well as gender, genetic susceptibility, and immunosuppression, still need clarification [4].

A high HHV8 seroprevalence in individuals engaging in high-risk sexual activity, including homosexual men, indicates the role of sexual behaviour in the transmission of the infection in adults [4]. On the other hand, the presence of HHV8 antibodies in subjects under the age of 16 and in nuns also suggests a non-sexual route of transmission, probably by saliva [4,8].

The HHV8 reservoir in peripheral blood appears to be mononuclear cells and its detection in mononuclear cells is predictive of KS development [9]. A sensitive method for detecting HHV8 in the mononuclear cells of individuals who subsequently develop KS is PCR. PCR is considered to be a specific and sensitive means of verifying KS in the differential diagnosis of angioproliferative lesions [10].

Clinically, the classic form of KS is restricted mainly to the surface of the body. Three distinct stages of progression, which can overlap, can be recognized: patch, plaque, and nodule. In the first stage, a clinically indistinct red-to-blue macule occurs, usually in the lower extremity. KS may be mistaken in the skin for an inflammatory dermatosis, pyogenic granuloma, angiodermatitis or pseudo-Kaposi's, bacillary angiomatosis, angiosarcoma, bullous lesion in the rare cases of lymphangioma-like or bullous KS, or arteriovenous malformations. In the case we have presented here, a raised, slightly pigmented skin lesion was initially regarded as a squamous cell carcinoma. The lesion's clinical presentation, its location, the otherwise normal skin, and the lack of any predisposing factors with the exception of the patient's origin, were misleading.

The best treatment modality is still a matter of debate and no standard treatment guidelines are available. Many patients have cutaneous lesions amendable to local therapy (cryotherapy, intralesional therapy, simple excision). Some patients require more aggressive local therapy (radiation therapy) or systemic therapies (interferon α, chemotherapy). Yet surgical excision has been frequently associated with local recurrence, while chemotherapy with vinblastin or bleomycin has been characterized as not very effective [11]. In contrast, there are indications that radiation therapy provides excellent palliation with minimal side effects [12], while there are indications that interferon α and IgG treatment can bring about regression [11,13]. It is recommended that KS be treated by high-speed electron radiation covering the lesion and adjacent sites of the intact skin. Total focal dose must not exceed 30 Gy and, in case of circulatory disturbances in the lower limbs, the dose should be reduced to 20 Gy [14]. Alternatively, a single application of 800 cGy may be as effective as a more protracted course of radiotherapy with a comparable complication rate [15]. Standardized staging criteria provide aid when deciding on appropriate local or systemic therapy and for assessing and comparing response therapies.

In our case, a wide local excision was performed because the lesion was regarded as a squamous cell carcinoma, for which standard surgical excision is the preferred treatment. If we had considered the possibility of KS, we would have chosen an incisional biopsy to determine the tumour histology. Excisional biopsy would not have been the preferable method because the lesion was too large to anticipate primary closure.

No matter what treatment modality is chosen, clinicians should bear in mind that even in its classical form, KS may be a malignant, rapidly progressing tumour with visceral involvement. Although primary hand KS is a relatively uncommon disorder in patients who are negative for HIV, dermatologists, venereologists, and surgeons should consider this possibility when treating non-specific lesions involving the extremities. Since various effective treatment options are available, watchful waiting is probably inappropriate in most cases.

## Conclusion

KS is an unusual vascular tumour that, similarly to other forms of cutaneous neoplasms, has a definite evolutionary course from a low-grade slow-growing early phase, to an anaplastic malignant neoplasm. Skin biopsy is important for making the correct diagnosis, with the added use of immunohistochemistry or molecular biology in equivocal cases.

Minimal hand lesions with non-distinctive clinical features may be the exclusive manifestation of KS, making clinical suspicion essential and histological evaluation necessary to establish the diagnosis.

## Abbreviations

HHV8: human herpes virus type 8; KS: Kaposi's sarcoma; PCR: polymerase chain reaction.

## Competing interests

The authors declare that they have no competing interests.

## Consent

Written informed consent was obtained from the patient for publication of this case report and any accompanying images. A copy of the written consent is available for review by the Editor-in-Chief of this journal.

## Authors' contributions

All the authors have contributed equally to this case report. All authors read and approved the final manuscript.
